# Few and Far Between: How HIV May Be Evading Antibody Avidity

**DOI:** 10.1371/journal.ppat.1000908

**Published:** 2010-05-27

**Authors:** Joshua S. Klein, Pamela J. Bjorkman

**Affiliations:** 1 Division of Biology, California Institute of Technology, Pasadena, California, United States of America; 2 Howard Hughes Medical Institute, California Institute of Technology, Pasadena, California, United States of America; The Fox Chase Cancer Center, United States of America

## HIV-1 Consistently Evades the Humoral Immune Response

More than 25 years have passed since the discovery of HIV type 1, the causative agent of AIDS, and the first vaccine candidate to exhibit evidence for protection against infection was reported only recently [1]. However, the extent and mode of protection are still under debate [2]. Thus, a vaccine that effectively stimulates complete protective immunity by the cellular branch (cytotoxic T lymphocytes) and/or the humoral branch (antibodies) of the immune system has yet to emerge. Among the millions of people who have received treatment for the disease and the many more who have tested HIV positive, there exists no definitive case in which a potent neutralizing antibody response enabled an infected individual to successfully clear or control the infection. In a small percentage of cases, individuals will exhibit a natural ability to suppress viral replication and progression of the disease. However, the explanation for the existence of this rare phenotype has primarily converged on a robust cellular immune response, with evidence generally lacking for a significant contribution to viral control by antibodies [3]–[5].

Structural features of the HIV envelope spike are critical to its unusual ability to escape neutralizing antibodies. However, many of the identified features are not unique to this virus. Here, we propose another strategy HIV employs to evade antibodies: the low density of envelope spikes, a distinguishing feature when compared with viruses to which protective neutralizing antibody responses are consistently raised, directly impedes bivalent binding by immunoglobulin G (IgG) antibodies. The result is a minimization of avidity, normally used by antibodies to achieve high affinity binding and potent neutralization, thereby expanding the range of mutations that allow HIV to evade antibodies. Understanding limitations to avidity may be essential to the design of anti-HIV vaccines and therapies.

## The HIV Spike Structure and Its Rapid Mutation Facilitate Antibody Evasion

Tremendous effort has been devoted to understanding why HIV so effectively evades antibodies. Accepted explanations include rapid mutation of the two glycoproteins that comprise the envelope spike, gp120 and gp41, and structural features that enable the spike to hide conserved epitopes from antibodies. These structural features include a shield of host-derived carbohydrates [6], conformational masking [7], steric occlusion [8], the protection of conserved regions at interfaces by oligomerization or in narrow pockets [9]–[11], and the presence of highly variable flexible loops that shield conserved epitopes on the envelope spike [9], [12]. In addition, it was recently hypothesized that a lack of germline genes capable of maturing into potent anti-HIV antibodies may represent holes in the potential antibody repertoire [13].

While the importance of the envelope spike's structural attributes to limiting antibody potency are well established, they are not unique to HIV. For example, the receptor binding sites of both rhinovirus and influenza are narrow pockets predicted to be inaccessible to antibodies [14], and mutation, loop decoys, and glycan shielding have all been implicated in antibody evasion by influenza [15], [16]. Nevertheless, these viruses and many others and/or the vaccines that have been developed against them elicit potent neutralizing antibody responses that significantly contribute to their clearance or provide sterilizing immunity [17].

What distinguishes HIV from other viruses in relation to antibody-mediated neutralization? Is it simply that HIV is more adept at employing the evasion strategies outlined above? While it is clear that HIV is superbly adapted for evading antibodies based on these strategies (as described in recent reviews [15], [18]), we propose an additional contributing factor in its ability to escape neutralization by antibodies [19], which is based on recent data that describe the spatial arrangement of spikes on its surface. The reasoning is rooted in an inherent limitation to the architecture of an antibody as it relates to avidity, which in this context refers to the ability of a bivalent antibody to simultaneously bind two epitopes tethered to the same surface [20]. We begin with comparisons of available neutralization data and the spatial arrangements of envelope spikes for HIV and other viruses, then present a discussion of avidity and the factors that influence it, and end with speculations on how a greater understanding of the factors that aid or inhibit avidity might be used to further inform vaccine design.

## Comparison of Monovalent and Bivalent Binding of Antibodies to Viruses

**Figure 1:**
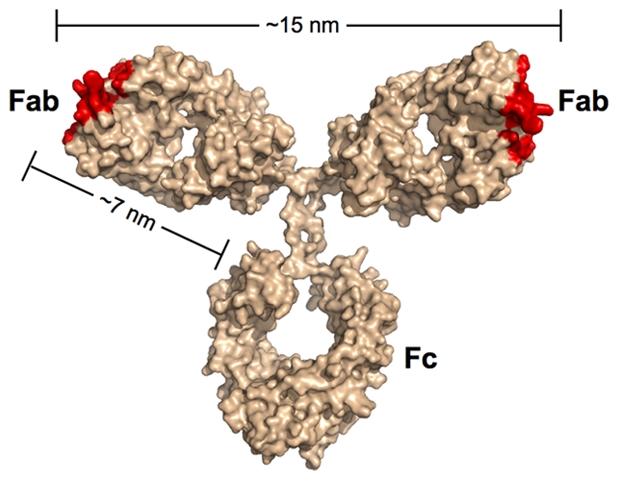
Scale model of an IgG antibody. Red denotes the locations of antigen recognition sites. A longer than typical separation distance (17 nm) was reported for the structure of intact b12 IgG [69]. The longer distance resulted in part from an unusually long CDR3 loop protruding from the antigen-combining site of each Fab. As this loop wraps around the CD4-binding loop on gp120 [64], the effective separation distance on this IgG and other antibodies with protruding CDR3 loops would be ∼15 nm.

Most of the neutralizing activity in the sera of HIV-positive individuals can be attributed to antibodies of the IgG subclass [21], [22], which represent the predominant class of immunoglobulin in blood. IgG antibodies are composed of an Fc region fused to two identical Fabs (Figure 1). Antigens bind to the tip of each Fab, which present the unique surfaces that define the epitope specificity of the antibody. While the immune system can draw from an almost unlimited sequence library to change the specificity of the Fabs, the antibody architecture is relatively constant, including the range of permissible end-to-end distances between the Fabs. The Fabs are linked to the Fc region by a flexible hinge, which typically allows a 10–15-nm center-to-center separation between the antigen-binding sites of the two Fabs for IgGs.

In the context of antibodies, the term avidity refers to their ability to bind two physically linked antigens simultaneously (e.g., to the surface of the same virus). The result of avidity can be a dramatic increase in the strength of the binding as compared to a monovalent 1∶1 interaction such that once bound, the antibody interaction with antigen becomes essentially irreversible over biologically relevant time scales [23]. Antibodies have been shown to bind bivalently to non enveloped viruses such as rhinovirus and poliovirus, which contain a rigid icosahedrally symmetric outer protein shell with closely spaced epitopes. Thus, rhinovirus and poliovirus saturate with 30 IgGs bound via both Fabs to 60 repeating epitopes created by 30 2-fold symmetry axes [24], [25]. An early demonstration of the importance of avidity in IgG binding to poliovirus revealed that using papain to digest the antibody and create monovalent Fabs led to a substantial increase in the molar concentration required to inhibit infection in vitro [25]. By contrast, a limited role for avidity in neutralization of HIV by some antibodies is suggested by the relatively modest increases in neutralization potencies of IgGs as compared to their corresponding Fabs [26]–[28]. In addition, conversion of the broadly neutralizing anti-HIV antibodies 2F5 and 4E10 from IgGs with two combining sites to dimeric IgA (four Fabs) and/or pentameric IgM (ten Fabs) either did not improve their neutralization efficiencies or abrogated activity altogether [29], [30]. Similarly, we have observed equivalent neutralization potencies for the anti-HIV antibody b12 when tested as an IgA, IgM, or IgG (P. Gnanapragasm, R. Galimidi, J. Klein, A. West, Jr., and P. Bjorkman, unpublished data).

**Figure 2:**
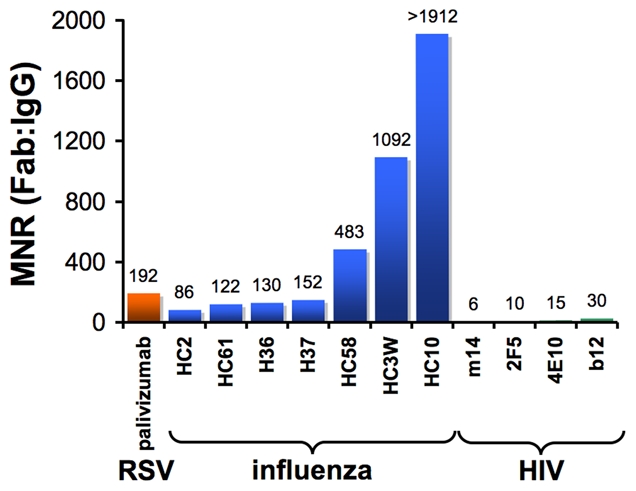
Bar graph of the highest reported molar neutralization ratios (MNRs). MNRs were reported for monoclonal antibodies against HIV [26], [27], [28], RSV [32], and influenza [31], [33]. The MNR for each antibody was calculated as the IC_50_ of the Fab divided by the IC_50_ of the IgG derived from in vitro neutralization assays (IC_90_s were reported for some influenza IgG/Fab comparisons [31], but IC_50_ ratios would be nearly the same because the slopes of the inhibition curves were similar). MNRs for a particular IgG/Fab combination can vary with the strain of virus being tested because the degree to which cross-linking can benefit an IgG depends on the affinity of the Fab for its antigen. Differences in size between a Fab and IgG may also influence the MNR if steric factors play a role in the neutralization mechanism of a particular antibody. However, this effect is probably minor, as (Fab)′_2_ fragments generally exhibit similar neutralization potencies to their parental IgGs [31], [33]. Not shown are high MNR values (∼70) derived for IgG/Fab comparisons involving HIV virions with a gp41 cytoplasmic tail truncation [70]. The tail deletion, which is rarely observed in vivo, has been suggested to increase the mobility of envelope trimers [70] and/or increase the number of spikes per virion [71], so its effects on intra-spike cross-linking are not well understood.

One way to quantitatively assess the effects of antibody avidity is to compare the neutralization potency values of a Fab and its parental IgG. We define the molar neutralization ratio (MNR) as the concentration in an in vitro neutralization assay at which a Fab achieves 50% inhibition of viral infectivity (IC_50_) divided by the IC_50_ for the parental IgG. If an antibody binds only monovalently to the viral surface (i.e., it is incapable of cross-linking epitopes on the virus), it would inhibit at an approximately 2-fold lower concentration than the Fab (MNR = 2) because the IgG has twice the number of antigen-binding sites [31]. MNRs greater than 2 suggest avidity effects resulting from the IgG cross-linking epitopes on the virus. Results from published studies show high MNRs for respiratory syncytial virus (RSV) [32] and influenza [31], [33] (Figure 2), suggesting that antibodies can take advantage of avidity effects to bind to enveloped viruses. However, a compilation of the highest reported MNRs we could find for antibodies against HIV [26]–[28] shows that neutralizing antibodies, including those that serve as models for the types of antibodies that researchers would most like to elicit with an HIV vaccine, yield relatively low MNRs (Figure 2). This suggests a general limitation to bivalent binding of IgGs to HIV. We propose that the spatial distribution of envelope spikes on HIV, combined with the distribution of protein epitopes on the spike trimer, explains the predominantly monovalent binding of anti-HIV antibodies, which in turn limits the ability of the humoral immune response to prevent viral escape by mutation.

## HIV Envelope Spikes Are Present at Low Density

**Figure 3:**
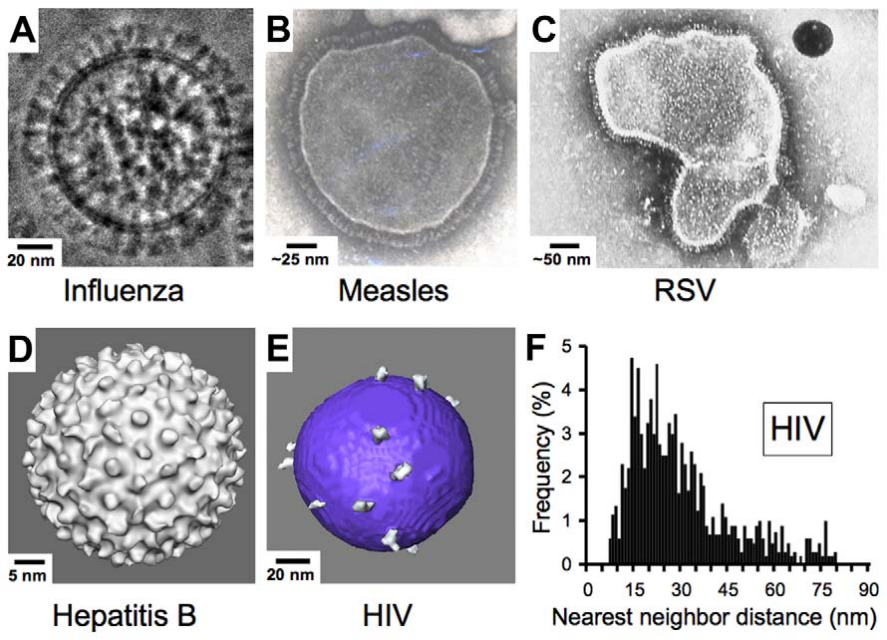
Comparison of enveloped viruses and nearest neighbor distances for HIV envelope spikes. (A) Influenza type A virus. Image provided by Drs. Masashi Yamaguchi and Kuniaki Nagayama. (B) Measles virus. Image reproduced with permission from Dr. Shmuel Rozenblatt from http://www.tau.ac.il/lifesci/departments/biotech/members/rozenblatt/figures.html. (C) RSV (image credit: US Centers for Disease Control and Prevention). (D) Hepatitis B virus. Image provided by Drs. Kelly Dryden and Mark Yeager. (E) HIV type 1. Image provided by Drs. Ping Zhu and Kenneth Roux. See also [38]. Many schematic pictures of HIV in textbooks and on Web sites show more spikes per virion. Some of these figures were based on early electron micrographs of a mutant simian immunodeficiency virus containing a higher number of spikes per viral particle [72]. Others were based on the incorrect assumption that HIV exhibits icosahedral symmetry. (F) Distribution of nearest neighbor distances between HIV spikes derived from cryo-ET analyses of 40 HIV virions. Data were taken from [38]. Although some spike clustering was reported [38], the virions exhibited a large distribution of nearest neighbor distances between spikes (7–80 nm center to center).

Enveloped viruses such as HIV contain an outer shell composed of a cell-derived lipid membrane displaying embedded antigens that were acquired during budding from the host cell. Consequently, enveloped viruses generally lack the structural elements of non-enveloped viruses that enforce a symmetric arrangement of antigens in non-enveloped viruses. Electron micrographs of enveloped viruses for which antibody-mediated neutralization is known to be critical to the control and/or elimination of infection [17], [34] generally reveal a high density of envelope spikes. For example, influenza type A virus incorporates ∼450 spikes per virus particle spaced at intervals ≤10 nm [35] (Figure 3A). Similarly, measles, RSV, and hepatitis B virions include large numbers of closely spaced spikes (Figure 3B–3D). Indeed, the high densities of repetitive, identical epitopes on the surfaces of non-enveloped icosahedral viruses and enveloped viruses such as vesticular stomatitis, rabies, influenza, and Sindbis allow induction of T cell–independent B cell activation during the elicitation of the humoral immune response [36]. In striking contrast, biochemical studies and cryo-electron tomography (cryo-ET) reconstructions showed that HIV, although similar in size to influenza type A, has an average of ∼14 spikes per virus particle (the full range from published studies is four to 35 spikes) [37]–[41] (Figure 3E). Despite the dearth of envelope spikes, HIV remains infectious, as it has been shown that as few as four spikes are sufficient for viral attachment [42], and possibly fewer may be needed to achieve fusion with the target cell membrane [43], [44].

## Low Spike Density and Spike Structure Impede Bivalent Binding by IgGs to HIV

What are the consequences of the low number of envelope spikes on HIV virions to antibody binding? Cryo-ET studies of HIV particles allowed an analysis of nearest neighbor distances between individual spikes, revealing that the low number of envelope spikes also translates to a low spike surface density. Thus, the majority of nearest neighbor distances fall outside of the range of the two Fabs of an IgG [38] (Figure 3F) as previously predicted [45], leaving a minority of HIV envelope spikes available for cross-linking by a bivalent antibody. Inter-spike cross-linking might still be possible if spikes were able to freely diffuse within the viral membrane, but analyses of cryo-ET data [38] and evidence for interactions between the cytoplasmic tail of gp41 and the matrix protein of HIV [46], [47] suggest that the arrangement of spikes on a virus particle is likely to be static over time periods relevant to neutralization.

Cross-linking within a spike trimer (intra-spike cross-linking) represents another way to achieve bivalent binding of an IgG. However, cryo-ET structures of HIV spike trimers bound to Fabs [39] and molecular modeling based on crystal structures [27], [48] suggest that bivalent binding within a single trimeric spike is also unlikely, at least for antibodies directed against gp41 or the CD4-binding site of gp120. Therefore, most anti-HIV antibodies probably bind only one epitope per spike. Anti-carbohydrate antibodies may be an interesting exception: since a single spike subunit contains many carbohydrate attachment sites, an anti-carbohydrate antibody can bind using both Fabs to adjacent carbohydrate sites within a spike monomer. Although antibodies that recognize viral carbohydrates are rare because viral carbohydrates are usually non-immunogenic, one broadly neutralizing antibody against HIV, IgG 2G12, presents its two Fabs as a single domain-swapped structure that recognizes a constellation of viral carbohydrates within gp120 [49] and appears to be unusually effective in conferring protection against infection in vivo [50]. A naturally occurring dimeric form of IgG 2G12 composed of four Fabs and two Fcs was recently found to exhibit a 100- to 160-fold average increased molar neutralization potency over its monomeric form, with an increase of ≥500-fold against seven of the 21 strains tested [51], suggesting an enhanced ability to cross-link carbohydrate epitopes on a single envelope spike. Another exception might be represented by a new class of highly potent and broadly neutralizing anti-HIV antibodies, which include PG9 and PG16 [52]. The location of the proposed epitope for these antibodies, at the top of the envelope spike, might allow both Fabs of a single IgG to bind the same spike trimer.

## How Avidity Can Enhance Antibody Potency: A Theoretical Examination

**Figure 4:**
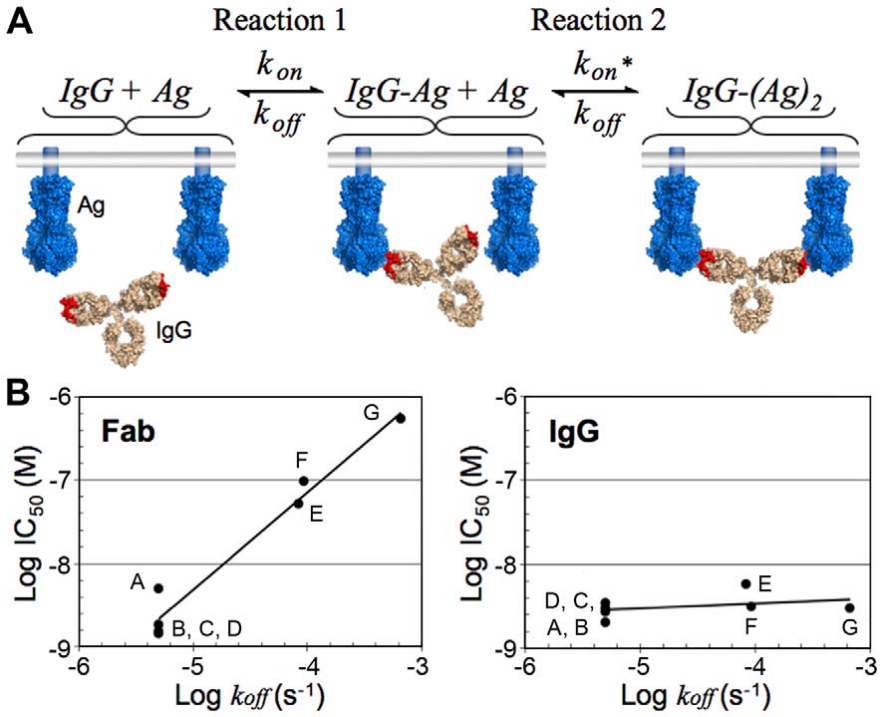
Bivalent binding model and effect of dissociation rate on neutralization in bivalent and monovalent binding. (A) Schematic of the step-wise bivalent binding model of an IgG to two envelope spikes (Ag, antigen) tethered to the same surface (*k_on_*, association rate constant; *k_off_*, dissociation rate constant; k_on_*, enhanced association rate constant resulting from the small reaction volume of Reaction 2). (B) Comparison of the effect of the Fab dissociation rate constant (*k_off_*) on the neutralization potency of Fab (left) and IgG (right) variants of palivizumab, a monoclonal antibody against RSV. Adapted from Table 1 in [32]. The names of the Fab/IgG pairs were changed to A–G for clarity. A, AFFFd; B, AFFYd; C, AFSFd; D, AFFGd; E, W100F; F, S32A; G, wild type (see [32] for an explanation of mutant nomenclature). Note that these results suggest that high affinity Fabs with slow dissociation rates (e.g., Fabs selected by techniques such as phage display) may not exhibit increased neutralization potencies, particularly against a densely packed virus, when converted to bivalent IgGs.

The affinity of a monomeric Fab, given by the equilibrium dissociation constant (*K_D_*), is equal to the dissociation rate constant (*k_off_*), divided by the association rate constant (*k_on_*). Overall, avidity manifests as an increase in the observed affinity of an IgG (a decrease in the *K_D_*) when binding two tethered antigens such that saturation of a surface can be achieved at lower concentrations as compared to a monovalent Fab. The affinity increase is mostly due to a reduction in the observed dissociation rate for the IgG such that binding to antigen becomes virtually irreversible over time scales relevant to the lifetime of a pathogen [23]. The effects of avidity on the affinity of an antibody can be modeled as a two-step reaction involving one antibody molecule and two epitopes tethered to the same surface (Figure 4A). After becoming tethered to its target through the first Fab, the small reaction volume of the second forward reaction serves to increase the second reaction rate [53], but this second binding step can only occur if the second binding site lies within the volume that is accessible to the free Fab arm. A corollary of the model for avidity is that the potency of a neutralizing IgG that can bind bivalently to two epitopes simultaneously on the same surface of a pathogen will primarily depend on the magnitude of *k_on_*. In contrast, the potency of a Fab or an IgG that binds monovalently will depend on the magnitudes of both the *k_on_* and the *k_off_*.

The predicted insensitivity of an IgG to changes in *k_off_* under conditions permissible to bivalent binding was demonstrated for palivizumab, a monoclonal antibody directed against RSV [32], an enveloped virus with a high spike density (Figure 3C). A comparison of neutralization potencies of Fabs and their parental IgGs for a library of antibody variants derived from palivizumab demonstrated that mutations that decreased *k_off_* did not change the potency of the corresponding IgG but did increase the neutralization potency of the Fab [32] (Figure 4B). Furthermore, as predicted by avidity effects, mutations that increased *k_on_* served to increase the neutralization potencies of both the Fab and the IgG [32]. Thus, for cases in which efficiently cross-linking the surface of a virus is likely (e.g., RSV or influenza), an antibody can maintain a relatively unchanged neutralization potency even as the virus accumulates mutations that increase its *k_off_*. However, in cases in which efficient cross-linking is unlikely (e.g., HIV), the virus can escape antibody-mediated neutralization with mutations that weaken either rate constant, resulting in a virus that can more easily escape the humoral immune system during the course of an infection.

## How Understanding Limitations to Avidity Can Inform the Design of Anti-HIV Vaccines and Therapies

The vertebrate immune system is remarkable in its ability to respond to and clear infections. Unfortunately, the relatively fixed distance between the two antigen-binding sites of an IgG and a reliance on avidity as a mechanism to achieve higher affinities makes it susceptible to evasion by pathogens that employ high mutation rates coupled with low antigen densities. When compared to the antigen densities present on the surfaces of viruses to which neutralizing antibody responses can be consistently raised (Figure 3A–3D), it seems an unlikely coincidence that HIV—a virus that is among the most adept at evading antibody-mediated neutralization—also stands out as having an unusually high mutation rate and an unusually low density of surface envelope spikes with apparently restricted mobility. Thus, it is tempting to speculate that whereas antibodies evolved to form a bivalent structure that enhances binding to pathogen surfaces through avidity effects, HIV evolved a low spike density designed to specifically thwart bivalent binding by antibodies.

In the initial immune response to a particular variant of HIV, it is likely that IgGs will exhibit sufficiently slow dissociation rates and high enough affinities to exert selective pressure even when binding monovalently, whether by neutralization of virus particles or by recruiting effector functions against infected cells. However, faced with a target to which bivalent binding is predominantly impossible, antibody potency will be susceptible to escape by a wider range of mutations: ones that serve to decrease the rate of association as well as ones that serve to increase the rate of dissociation. The immune system may respond with revisions to the antibody repertoire, but the rate at which new antibodies are made will be easily outpaced by the virus's rate of mutation.

Without the buffering effect against escape by mutation that avidity provides, it is likely that immunogens derived from HIV will need to be specifically tailored to focus the antibody response against only the most conserved epitopes—a key objective that has already been identified by many research groups [54], [55]. Viewed through the lens of avidity considerations, a general deficiency in bivalent binding will impose the additional requirement that broadly neutralizing antibodies still exhibit high affinities for their epitopes when binding monovalently. An alternative approach, as others have proposed [56], [57], may lie in eliciting anti-carbohydrate antibodies, as the high density of glycans on each gp120 monomer should enable efficient bivalent binding to individual envelope spikes. Thus, new immunogens designed to elicit antibodies capable of intra-spike cross-linking—either carbohydrate epitopes within or between spike monomers, or protein epitopes between spike monomers—may prove critical to the induction of a broadly cross-reactive neutralizing antibody response.

Using available crystallographic [9], [58]–[66] and electron microscopy data [35], [37]–[39], [67], [68], it might also be possible to engineer novel bivalent and multivalent antibody architectures that are capable of intra-spike cross-linking by increasing the reach between Fabs using insertions in the hinge region of an IgG that adopt extended conformations [19], although they would need to be administered via passive immunization or gene therapy. Carbohydrate-binding reagents specific for HIV (perhaps based on the anti-carbohydrate antibody 2G12) might be a logical starting point, as multimerization of 2G12 has been shown to significantly enhance its neutralization potency [30], [51]. These engineering approaches, as well as the design of immunogens able to elicit intra-spike cross-linking antibodies, could hold a significant advantage in that either approach would make the low spike density on HIV irrelevant to neutralization potency.
